# RETRACTION: Clinical Study on the Role of lncRNA STX17‐AS1 in Wound Healing and Hypertrophic Scar Formation

**DOI:** 10.1111/iwj.70492

**Published:** 2025-04-02

## Abstract

**RETRACTION:** WangW. , LiuM. , LiX. , LiuG. , and WangC. , “Clinical Study on the Role of lncRNA STX17‐AS1 in Wound Healing and Hypertrophic Scar Formation,” International Wound Journal 21, no. 2 (2024): e14658, 10.1111/iwj.14658.PMC1083090942052922

The above article, published online on 31 January 2024, in Wiley Online Library (http://onlinelibrary.wiley.com/), has been retracted by agreement between the journal Editor in Chief, Professor Keith Harding; and John Wiley & Sons Ltd. Following an investigation by the publisher, all parties have concluded that this article was accepted solely on the basis of a compromised peer review process. In addition, the authors provided incomplete ethical approval and patient consent information for the study. The editors have therefore decided to retract the article. The authors did not respond to our notice regarding the retraction.

**RETRACTION:** W. Wang , M. Liu , X. Li , G. Liu , and C. Wang , “Clinical Study on the Role of lncRNA STX17‐AS1 in Wound Healing and Hypertrophic Scar Formation,” International Wound Journal 21, no. 2 (2024): e14658, 10.1111/iwj.14658.PMC1083090942052922

The above article, published online on 31 January 2024, in Wiley Online Library (http://onlinelibrary.wiley.com/), has been retracted by agreement between the journal Editor in Chief, Professor Keith Harding; and John Wiley & Sons Ltd. Following an investigation by the publisher, all parties have concluded that this article was accepted solely on the basis of a compromised peer review process. In addition, the authors provided incomplete ethical approval and patient consent information for the study. The editors have therefore decided to retract the article. The authors did not respond to our notice regarding the retraction.

